# *Praecitrullus fistulosus* Extract Exhibits Antidiabetic Potential by Augmenting Insulin-Signaling Cascade, GLUT-4 and IRS-1, in Streptozotocin–Nicotinamide-Induced Diabetic Rats

**DOI:** 10.3390/foods14213764

**Published:** 2025-11-03

**Authors:** Ayesha Amjad, Azmat Ullah Khan, Qaisar Raza, Sajid Khan Tahir

**Affiliations:** 1 Department of Food Science & Human Nutrition, University of Veterinary and Animal Sciences, Outfall Road, Lahore 54000, Pakistan; aisha_amjaad@yahoo.com (A.A.); qaisar.raza@uvas.edu.pk (Q.R.); 2 Department of Physiology, University of Veterinary and Animal Sciences, Outfall Road, Lahore 54000, Pakistan; sajid.tahir@uvas.edu.pk

**Keywords:** *P. fistulosus* extract, insulin-related indices, antioxidants, gene expression, protein expression

## Abstract

Diabetes mellitus is largely driven by oxidative stress that disrupts insulin signaling, leading to failure in insulin-mediated glucose absorption. Exploration of natural bioactive compounds is fueled by their promising role in correcting redox imbalance. This study aims to investigate the antidiabetic effect of the methanolic extract of *Praecitrullus fistulosus*, potentially by transcriptional modulation in streptozotocin–nicotinamide-induced diabetic rats. Male Wistar albino rats (*n* = 36) were assigned to six groups: normal control; diabetic control; standard drug group; and three treatment groups receiving *P. fistulosus* extract orally at doses of 200, 400, and 600 mg/kg body weight, respectively, for 30 consecutive days. Diabetes was induced in all groups, except for normal control, by intraperitoneal co-administration of streptozotocin and nicotinamide. Nicotinamide (100 mg/kg) was injected 15 min prior to a single dose of streptozotocin (50 mg/kg). Baseline and endpoint assessments of weight and blood glucose levels were performed. Blood was processed to assess insulin-related indices, lipid profile, and oxidative stress markers. q-PCR and Western blotting were utilized to explore the underlying molecular mechanisms. The diabetic control-group rats exhibited impaired glucose tolerance due to the marked reduction in serum insulin levels, compromised β-cell function, and substantial rise in lipid profile and oxidative stress parameters. Oral administration of *P. fistulosus* methanolic extract effectively mitigated these alterations in a dose-dependent manner, accompanied by the upregulation of both gene and protein expression involved in the insulin-signaling cascade.

## 1. Introduction

In 2019, diabetes accounted for 4.2 million deaths worldwide [1]. It is ranked as the fourth leading cause of disability, establishing it as a global health emergency. With approximately 33 million affected individuals, Pakistan holds the third-highest global burden of diabetes [2]. Over 90% of diabetes cases are classified as type 2 diabetes mellitus (T2DM), characterized by insulin resistance and relative insulin deficiency that disrupts glucose uptake by tissues, resulting in intracellular hypoglycemia and extracellular hyperglycemia. Epidemiological studies suggest that diabetic mortality is largely attributed to vascular complications rather than hyperglycemia alone [3]. Oxidative stress induced through glucose oxidation, non-enzymatic protein glycation, and elevated lipid peroxidation levels overwhelm the enzymatic antioxidant defense systems. It significantly contributes to endothelial dysfunction, a key factor in the development of macro- and microangiopathies. Concurrently, a high concentration of glucose, insulin, and serum lipids not only exacerbates the vascular damage but also accelerates the atherosclerosis progression [4] and insulin resistance [5].

The current healthcare practices have proven to be insufficient in managing T2DM due to the disease’s multifactorial etiology and heterogeneous clinical manifestations. Recent studies are centered on enhancing the bioavailability and therapeutic effectiveness of the phytochemicals, highlighting their potential as substitutes for traditional pharmaceutical therapies [6]. Among the Cucurbitaceae family, *Praecitrullus fistulosus* is an endemic vegetable of the South Asian region, particularly cultivated in India, Pakistan, and Afghanistan. It is widely known as round melon, apple gourd, or Indian pumpkin, and is natively called tinda. Apart from being candied and pickled, it is also consumed as a cooked vegetable in Pakistani cuisine [7]. Oral administration of methanolic extract of *P. fistulosus* at dosages of 100 and 200 mg/kg body weight (b.w.) has indicated DNA-protectant potential and hepatoprotective activity in CCl_4_-mediated hepatic injury [8], in addition to its anti-helminthic activity against *Pheretima posthuma* [9]. The therapeutic potential of *P. fistulosus* is owed to its noteworthy bioactive compounds, like flavonoids, alkaloids, saponins, tannins, phytosterols, diterpenes, glycosides, and phenols [10].

A novel carbohydrate-binding protein named lectin, isolated from the fruit sap of *P. fistulosus*, revealed itself to have a promising anti-proliferative effect in various cancer cell lines, along with its in vivo anti-angiogenic potential, by inhibiting metalloproteinases activity without eliciting any significant detrimental effects in normal mice [11]. An earlier report has suggested that the ethanolic extract of *P. fistulosus* at a dose of 300 mg/kg b.w. exhibits antioxidant and antidiabetic potential in streptozotocin-induced diabetic rats [12]. Nevertheless, the underlying pathways involved in *P. fistulosus* extract’s action are not well explored. In the streptozotocin–nicotinamide (STZ-NA) model of T2DM, NA provides partial protection to pancreatic β-cells against the cytotoxic effects of STZ [13]. Consequently, a state marked by moderate, stable hyperglycemia with 40% reduction in β-cell mass and substantial depletion (60%) of pancreatic insulin reserves is established, which exhibits diabetes-related symptoms, including polyphagia and polydipsia [14]. To date, the literature lacks studies that provide detailed mechanistic insight into the antidiabetic potential of the *P. fistulosus* through gene expression and protein profiling using real-time PCR and Western blotting. Hence, to address this research gap, the present investigation evaluates the antihyperglycemic effect of *P. fistulosus* methanolic extract against STZ-NA-induced diabetic rats compared to the standard drug (metformin).

## 2. Materials and Methods

### 2.1. Chemicals and Reagents

Analytical-grade chemicals and reagents were utilized in this study. Streptozotocin and nicotinamide were purchased from Sigma-Aldrich Co. (St. Louis, MO, USA). Metformin (Abbott Laboratories Pak Ltd., Karachi, Pakistan) was procured from a local pharmacy in Lahore, Pakistan. Blood glucose levels were monitored using the Accu-Chek Performa^®^ (Roche Diagnostics Pakistan Ltd., Karachi, Pakistan) blood glucose meter. Rat insulin ELISA assay kit was acquired from Sigma-Aldrich Co. (St. Louis, MO, USA). Analytical kits for lipid profile (total cholesterol, triglycerides, and HDL) were acquired from Diagnostic System-SIEMENS, Response^®^ 910, Munich, Germany. The PCR workflow involved TRIzol-based RNA isolation (Ambion, Thermo Fisher Scientific, Waltham, MA, USA), cDNA synthesis using SuperScript III (Invitrogen, Thermo Fisher Scientific, Waltham, MA, USA), and amplification with SYBR Green Supermix (Bio-Rad, Hercules, CA, USA) on the MyiQ2 Real-Time PCR system (version 2.1.97).

### 2.2. Plant Authentication

Whole *P. fistulosus* fruit was procured during the mid-summer season from a local market in Lahore, Punjab, Pakistan. The species was authenticated from the Botany department, G.C University, Lahore, Pakistan, with an identification number (GC. Herb. Bot. 3974).

### 2.3. Extract Preparation

Immature green fruits of *P. fistulosus*, commonly used in culinary and medicinal applications, were harvested. They were peeled, diced, and then air-dried under shade (35 °C) for 7 days. The dried samples were then ground to a fine consistency. This powder was placed in a shaking incubator suspended in 98% methanol (1:10 *w*/*v*) at room temperature and agitated at 120 rpm for 72 h. Subsequently, the extract was filtered through filter paper (Whatman No. 1). Any excess solvent was evaporated using a rotary evaporator (Hei-VAP Value, Heidolph, Schwabach, Germany) at 50 °C. The concentrated crude extract was preserved at 4 °C for future analysis.

### 2.4. Laboratory Animals

Thirty-six male Wistar albino rats weighing 185–195 g, aged 6 weeks, were purchased from the animal facility of the Faculty of Biosciences, University of Veterinary and Animal Sciences, Lahore, Pakistan. The animals were kept under standard conditions of a 12 h light/dark cycle at 24 ± 2 °C. The rats had access to fresh water and a standard rat diet. This study was carried out following the approval granted by the ethical review committee of the University of Veterinary and Animal Sciences, Lahore (Reg No. DR/158; April 2023).

### 2.5. Induction of Diabetes

Diabetes was induced in all groups, except for the normal control, via intraperitoneal co-administration of streptozotocin and nicotinamide. Nicotinamide (100 mg/kg) dissolved in saline was injected 15 min prior to a single dose of streptozotocin (50 mg/kg) (STZ reconstituted in 0.01 M citrate buffer with pH 4.5) [13]. The normal control group received an equal dose of the citrate buffer. Blood glucose levels were monitored after 72 h using a glucometer. Rats with fasting glucose levels ≥ 200 mg/dL one week post-STZ induction were considered diabetic and included in further experimentation.

### 2.6. Experimental Design

After a 7-day acclimation period, the rats were assigned to six groups, with each group consisting of six rats. The treatments and metformin were administered via oral gavage once daily. The rat groups were constituted as follows: Group I (normal control group) received normal saline only; Group II (diabetic control group), diabetic rats received normal saline; Group III (standard drug group), diabetic rats treated with 100 mg/kg body weight (b.w.) of metformin dissolved in distilled water; Group IV (PFE 200), diabetic rats treated with 200 mg/kg b.w. of *P. fistulosus* methanolic extract; Group V (PFE 400), diabetic rats treated with 400 mg/kg b.w. of *P. fistulosus* methanolic extract; and Group VI (PFE 600), diabetic rats treated with 600 mg/kg b.w. of *P. fistulosus* methanolic extract. At the end of the trial, rats were euthanized under sodium pentobarbital anesthesia, following an overnight fast. Blood was drawn through cardiac puncture and processed to quantify serum biochemical markers. Pancreases were excised and processed for histology. Additionally, both pancreas and serum were preserved in an ultra-low-temperature freezer (at −80 °C) for further analysis.

### 2.7. Body Weight

The body weight of rats in all experimental groups was measured at the beginning and end of the study, with the average weight calculated for each group.

### 2.8. Fasting Blood Glucose Level

Blood glucose levels (FBG) were monitored at the beginning and end of the study period, under fasting conditions, via the tail-vein blood collection method, using a glucometer, as per the guidelines [15].

### 2.9. Oral Glucose-Tolerance Test

An oral glucose-tolerance test (OGTT) was performed during the 4th week of the 30-day trial. The FBG (0 min) was measured after an overnight fast of 12 h. Subsequently, rats received 2 g/kg b.w. of glucose via oral gavage. Blood glucose levels were periodically monitored at 30, 60, and 120 min intervals after administration via the tail pricking method using, a glucometer. Area under the curve (AUC) was estimated by GraphPad Prism version 10.2.0 (GraphPad Software, MA, USA), as described in the literature [16].

### 2.10. Serum Insulin Levels and Insulin-Related Index

Fasting serum insulin levels were assessed on day 30 using an ELISA kit, following the manufacturer’s standard protocol. Homeostatic Model Assessment of Insulin Resistance (HOMA-IR), insulin sensitivity index, and β-cell function index were estimated based on FBG levels and fasting serum insulin levels, using HOMA 2 calculator version 2.2.3 [17].

### 2.11. Relative Organ Weight

Organs (kidney, heart, and pancreas) were excised and washed in ice-cold saline. Relative organ weight was calculated as the percentage of organ weight relative to the terminal body weight of each rat:

$$Relative\;Organ\;Weight=\frac{Organ\;weight\;(g)}{Body\;weight\;(g)}\;\times 100$$

### 2.12. Lipid Profile Determination

Serum was separated via the centrifugation of blood samples at 3500 g, maintained at 4 °C for 20 min. Biochemical quantification of lipid profile, including total cholesterol (TC), triglycerides (TGs), and high-density lipoprotein (HDL), was performed with commercial kits, using blood chemistry analyzer (Response^®^ 910, Diagnostic System-SIEMENS, Munich, Germany) as per supplier instructions. Low-density lipoprotein (LDL) was estimated by Friedewald’s equation [18], and very low density lipoprotein (VLDL) was calculated using the equation from [19], as stated below:

 LDL=[TC−(HDL+(TG/5))]

$$VLDL=[TC-\left(HDL+LDL\right)]$$

### 2.13. Antioxidant Parameters

Degree of lipid peroxidation in serum was assessed colorimetrically by determining thiobarbituric acid reactive substances (TBARSs) in supernatant at a 490 nm wavelength, following the method by [20], and expressed as malondialdehyde (MDA). Catalase (CAT) activity in serum was determined by Reference [21]’s method. Absorbance was measured at a 374 nm wavelength thrice at 3 min intervals.

### 2.14. Serum TNF-α and IL-6 Levels

TNF-α and IL-6 in serum were estimated by using ELISA kits (Invitrogen, Thermo-SCIENTIFIC, Rockford, IL, USA), following the manufacturer’s standard protocol.

### 2.15. Isolation of RNA and Gene Expression Quantification in Skeletal Muscles Using Real-Time PCR

After the sacrifice, the skeletal muscles of rats were excised, followed by washing with normal saline, and then the skeletal muscles were kept in an ultra-low-temperature freezer at −80 °C. TRIzol reagent (Ambion, USA) was used for total RNA extraction from the frozen tissues. Total RNA concentration and purity were quantified using a NanoDrop spectrophotometer at an absorbance of 260 nm. Following the manufacturer’s instructions, 1 µg of RNA was reverse-transcribed into complementary DNA (cDNA) using SuperScript III Reverse Transcriptase (Invitrogen, Villebon, France) in a 20 µL reaction mixture. The cDNA synthesis involved denaturation at 70 °C for 5 min, followed by elongation at 55 °C for 45 min. MyiQ2 RealTime-PCR (Bio Rad, Marnes-la-coquette, France) was utilized for PCR using SYBR Green (Bio Rad) Supermix for 45 cycles of 95 °C for 30 s, 60 °C for 30 s, and 72 °C for 30 s, keeping the GAPDH as a housekeeping gene. Primer 3 website was used to design primers of GLUT-4 and IRS-1 genes that were analyzed for the antidiabetic model (Table 1). Fluorescence was measured in real-time during the reaction, enabling continuous tracking of PCR product formation.

### 2.16. Western Blotting

Western blotting was performed using standard procedures [22]. Frozen skeletal muscles tissues were weighed and then thawed on ice to prepare tissue homogenates in phosphate buffer saline (PBS), pH 7.0. The cell lysate was clarified by centrifugation at 3000 rpm for 10 min at 4 °C. The resulting supernatant was lysed in 500 μL of RIPA buffer (Thermo-scientific) using a tissue lyzer at 25 Hz. Cellular debris was removed from crude lysate via centrifugation (12,000 rpm, 20 min, 4 °C), and the resulting supernatant was collected as the whole-cell lysate. The Bradford assay (Bio-Rad Laboratories, Inc.) estimated the protein concentrations in each fraction by measuring absorption at 562 nm, using a microplate reader.

Equivalent amounts of protein (30 µg) were suspended with 2× Laemmli buffer, subjected to boiling for 5 min, and fractionated on 12% SDS-PAGE gels (Bio-Rad Mini-PROTEAN^®^). Proteins were blotted onto polyvinylidene fluoride PVDF membrane (Bio-Rad Immun-Blot^®^ PVDF Membrane) using wet transfer at 100 V for 90 min, with Precision Plus Protein™ Unstained Standards serving as a molecular-weight marker. Immunoblots were probed for 16 h at 4 °C with primary antibodies: anti-IRS-1, IRS1 Polyclonal Antibody (Invitrogen^TM^ (1:500)), and anti-GLUT-4 (Sigma-Aldrich Co. (St. Louis, MO, USA (1:1000)), with anti-β-actin (Bio-Rad Precision Ab β-Actin Antibody (1:4000)) serving as a loading control. Subsequently, the membrane was incubated with horseradish peroxidase (HRP)-conjugated secondary antibody (Thermo-Scientific Goat anti-Rabbit IgG-HRP (1:4000)) diluted in blocking buffer (5% skimmed milk in TBST) for 1 h at room temperature. Bands were visualized using an enhanced chemiluminescence (ECL) detection system and imaged using the ChemiDoc XRS+ system (Bio-Rad, Hercules, CA, USA) with Image Lab Software version 6.1. The intensity of each protein band was quantified and normalized to β-actin to account for loading variations. Densitometric quantification was performed using ImageJ software version 1.43 (NIH, Bethesda, MD, USA).

### 2.17. Histopathological Assessment

Once rats were sacrificed, their pancreas pieces were rinsed with saline and then fixed with 10% formalin. Afterward, tissues were gradually dehydrated using a sequence of ethanol solutions, cleared in xylene, and then embedded in paraffin wax. The pancreatic tissues were cut into precise sections of 5 μm thickness, followed by staining with H&E (hematoxylin and eosin) dye. Tissue morphology was subsequently examined under microscope (Olympus CX 31 with DP 20 software version 2.2) to identify histopathological changes.

### 2.18. Statistical Analysis

Data were analyzed using SPSS version 26 (Statistical Package for Social Sciences; SPSS Inc., Chicago, IL, USA). Results were presented as mean ± standard deviation (S.D.). Group differences were analyzed using one-way ANOVA, with Tukey’s post hoc test applied for pairwise comparisons. A *p*-value of less than 0.05 was regarded as significant.

## 3. Results

### 3.1. Effect of P. fistulosus Extract on Body Weight

Body weights of rats were recorded at the baseline (0th day) and at the end of the 30-day diabetes efficacy trial, as shown in Table 2. The normal control group showed a significant weight gain of 23.54%. In contrast, the diabetic control group exhibited significant weight loss (18%) compared to its baseline value, indicating the progression of the diabetic condition. The standard drug group showed a slight increase in body weight, suggesting a modest protective effect. Rats treated with *P. fistulosus* extract at doses of 200, 400, and 600 mg/kg exhibited dose-dependent weight stabilization, with the 600 mg/kg dose producing the most pronounced effect.

### 3.2. Effect of P. fistulosus Extract on Fasting Blood Glucose

Compared to the normal control group, the diabetic control group showed a significant increase in fasting blood glucose levels, rising from 289 ± 4.21 mg/dL at baseline to 300 ± 7.37 mg/dL on day 30, indicating severe hyperglycemia. As presented in Table 3, the standard drug group showed a significant reduction in blood glucose levels (*p* < 0.05). However, the *P. fistulosus* extracts showed a dose-dependent reduction in fasting glucose levels to 209 ± 3.52 mg/dL and 175 ± 2.77 mg/dL in the 200 and 400 mg/kg groups, respectively (*p* < 0.05; Table 3). Notably, the 600 mg/kg dose of *P. fistulosus* extract elicited the most significant response, reducing glucose levels from 280 ± 3.71 mg/dL to 138 ± 2.41 mg/dL by day 30 (*p* < 0.05). These findings support the antihyperglycemic effect of *P. fistulosus* extracts in comparison to the diabetic control group.

### 3.3. Effect of P. fistulosus Extract on Oral Glucose-Tolerance Test (OGTT)

**Figure 1:**
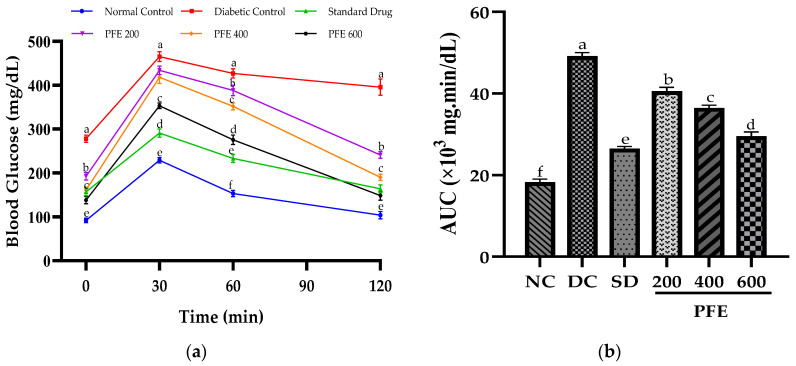
Effect of *P. fistulosus* extract on (**a**) changes in blood glucose levels in the glucose-tolerance test. (**b**) Area under the curve of blood glucose levels. Mean ± S.D. values were calculated using data from six rats per group. Bars labelled with distinct letters show a significant difference (*p* < 0.05). SD: standard drug. PFE: *P. fistulosus* extract.

On day 29 of the experimental trial, an OGTT test was performed following the intragastric administration of glucose at a dose of 2 g/kg body weight (b.w.). As shown in Figure 1a, the blood glucose level (BGL) of the rats exhibited a rapid increase within 30 min, followed by a gradual decline, reflecting the glucose-tolerance profile of each experimental group. The diabetic control group with baseline BGL (278 ± 4.6 mg/dL) demonstrated the highest peak at 30 min (465 ± 7.5 mg/dL), and this peak persisted at 60 min (427 ± 5.1 mg/dL) and remained elevated even at 120 min (395.5 ± 6.3 mg/dL), indicating severe glucose intolerance. Administration of *P. fistulosus* extract at 600 mg/kg significantly improved glucose tolerance by mitigating abnormal elevation in BGL at 30 min and facilitating a return to near-baseline levels by 120 min, similar to the response observed in the standard drug group. In contrast, *P. fistulosus* extract at doses of 200 and 400 mg/kg exhibited a rapid spike in BGL at 30 min, followed by a decline, but levels remained elevated at 120 min, indicating persistent glucose intolerance.

The area under the curve (AUC) for the OGTT represents the total glycemic load over time and serves as a comparative measure of glucose tolerance across the groups. The diabetic control group exhibited the highest AUC (49,200 mg·min/dL), significantly higher than that of the normal control (18,255 mg·min/dL), confirming severe glucose intolerance (*p* < 0.05). However, the AUC values for the treatment groups were notably lower: standard drug group (26,490 mg·min/dL), PFE 200 (40,595 mg·min/dL), PFE 400 (36,495 mg·min/dL), and PFE 600 (29,532 mg·min/dL), indicating a dose-dependent improvement in glucose clearance.

### 3.4. Effect of P. fistulosus Extract on Serum Insulin Levels

**Figure 2:**
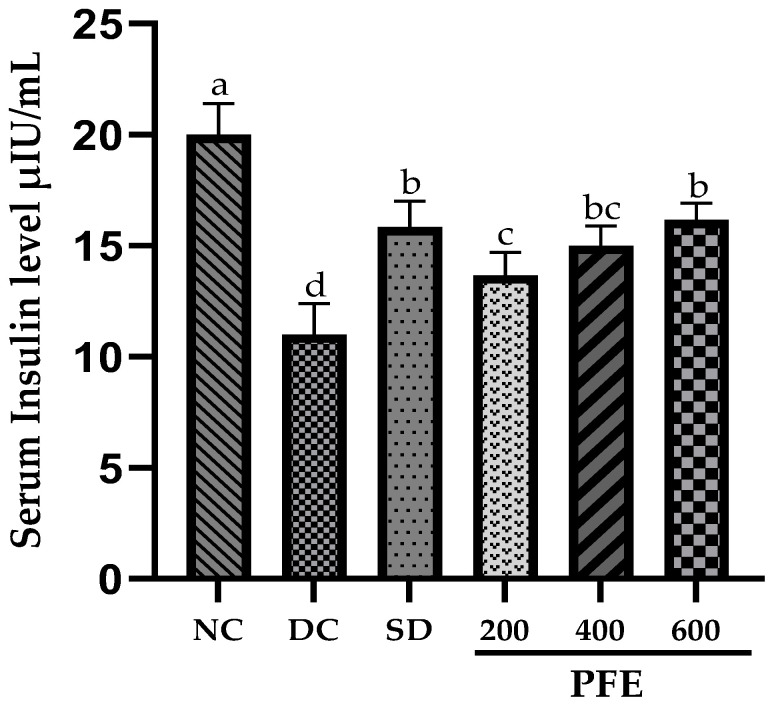
Effect of *P. fistulosus* extract on serum insulin levels in STZ-NA-induced diabetic rat model. Mean ± S.D. values were calculated using data from six rats per group. Bars labelled with distinct letters show a significant difference (*p* < 0.05). SD: standard drug. PFE: *P. fistulosus* extract.

Induction of diabetes with streptozotocin and nicotinamide significantly reduced (*p* < 0.05) the serum insulin levels in the diabetic control group in comparison to the normal control group. Co-administration of *P. fistulosus* extract at a dose 200 mg/kg resulted in a moderate but significant improvement (*p* < 0.05) in serum insulin levels, whereas the 400 and 600 mg/kg demonstrated significantly improved insulin levels, comparable to the standard drug (Figure 2).

### 3.5. Effect of P. fistulosus Extract on Insulin-Associated Index

Insulin resistance (IR), β-cell function (%B), and insulin sensitivity (%S) were estimated from fasting blood glucose levels and serum insulin levels (Table 4). Assessment of the insulin-associated index revealed that HOMA-IR values remained comparable across all the groups, suggesting a lack of insulin resistance. However, the diabetic control group reported pronounced reduction in β-cell function (%B: 17.3 ± 0.63), confirming the onset of diabetes. Treatment with *P. fistulosus* extract significantly improved insulin sensitivity and β-cell function in a dose-dependent manner. Notably, the 600 mg/kg dose significantly attenuated HOMA-IR to 2.44 ± 0.04 (*p* < 0.05 vs. diabetic control) and restored %B to 75.1 ± 0.42 and %S to 41.6 ± 0.25, achieving levels comparable to those observed in the standard drug group.

### 3.6. Effect of P. fistulosus Extract on Relative Organ Weight

Relative organ weight is a critical marker to monitor the progression of diabetes-associated complications. Our analysis revealed that relative kidney and heart weights remained statistically unchanged across all experimental groups, including the diabetic control in comparison to the normal control. In contrast, the pancreas weight was significantly reduced in the diabetic control (0.44 ± 0.01%) relative to the normal control group (0.64 ± 0.05), indicating atrophy or apoptosis. Treatment with *P. fistulosus* extract at 400 and 600 mg/kg restored pancreatic weight (0.58 ± 0.02% and 0.60 ± 0.08%, respectively), comparable to the standard drug group (0.61 ± 0.03%), as shown in Table 5.

### 3.7. Effect of P. fistulosus Extract on Lipid Profile

The diabetic control group showed significant dyslipidemia (*p* < 0.05), characterized by elevated TG (159 ± 3.4 mg/dL), TC (173 ± 3.3 mg/dL), LDL (125 ± 4.4 mg/dL), and VLDL (32 ± 2.4 mg/dL), alongside decreased HDL (16 ± 1.5 mg/dL) levels. Treatment with both metformin and *P. fistulosus* extracts significantly ameliorated lipid parameters. Among the extract-treated groups, PFE 600 significantly reduced TG (121 ± 2.1 mg/dL), TC (125 ± 1.8 mg/dL), LDL (72 ± 1.8 mg/dL), and VLDL (24 ± 2.1 mg/dL), while increasing HDL (29 ± 1.2 mg/dL). PFE at 400 mg/kg also showed notable improvements, particularly in lowering LDL (88 ± 1.1 mg/dL) and VLDL (25 ± 1.8 mg/dL, *p* < 0.05), as shown in Table 6. These findings indicate that *P. fistulosus* extract exhibits a dose-proportional antihyperlipidemic response.

### 3.8. Effect of P. fistulosus Extract on MDA and Catalase

Diabetic rats exhibited the highest MDA levels (2.90 ± 0.15 μmol/L) and the lowest catalase activity (0.40 ± 0.08 KU/L), indicating severe oxidative stress (Table 7). Treatment with metformin and *P. fistulosus* extracts reduced MDA levels and restored catalase activity in a dose-dependent manner. Among the *P. fistulosus* extract groups, the PFE 600 group showed an MDA reduction to 1.40 ± 0.06 μmol/L and a notable rise in catalase activity (1.51 ± 0.11 KU/L), nearly matching the values of the normal control group. These findings highlight the antioxidant potential of *P. fistulosus* extract, particularly at higher doses.

### 3.9. Effect of P. fistulosus Extract on TNF-α and IL-6

Serum levels of the proinflammatory cytokines TNF-α (161 ± 6.20 pg/mL) and IL-6 (93.33 ± 4.63 pg/mL) were significantly elevated (*p* < 0.05) in the diabetic control group. However, all treatments have shown a marked reduction (*p* < 0.05) in the proinflammatory cytokine levels, with PFE 600 exhibiting the greatest efficacy in mitigating the oxidative stress, as demonstrated in Table 8.

### 3.10. Effect of P. fistulosus Extract on GLUT-4 and IRS-1 Expressions in Skeletal Muscles

**Figure 3:**
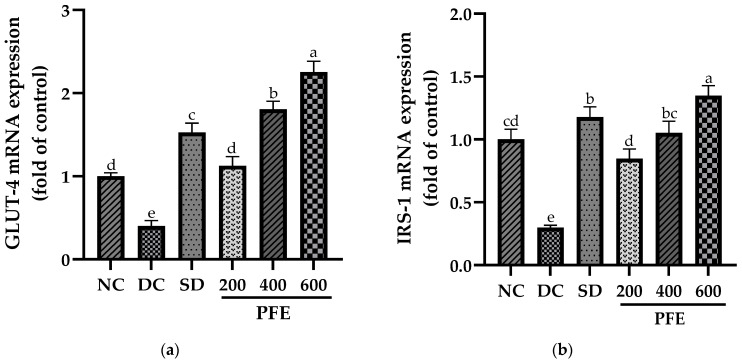
Effect of *P. fistulosus* extract on (**a**) GLUT-4 mRNA expression and (**b**) IRS-1 mRNA expression in skeletal muscles in STZ-NA-induced diabetic rat model. Data are expressed as mean ± S.D. values, calculated using data from three independent experiments per group. Bars labelled with distinct letters show a significant difference (*p* < 0.05). SD: standard drug. PFE: *P. fistulosus* extract.

**Figure 4:**
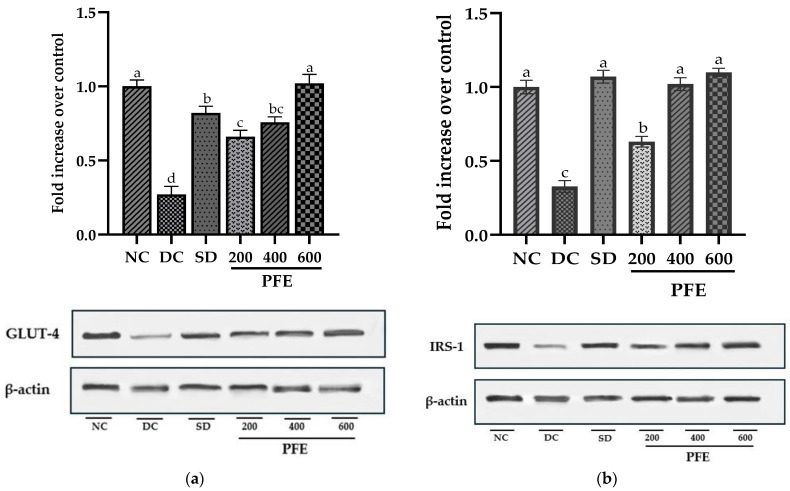
Effect of *P. fistulosus* extract on (**a**) protein expression and densitometric analysis of GLUT-4, and (**b**) protein expression and densitometric analysis of IRS-1, with β-actin serving as a loading control, as depicted in Western blots in the skeletal muscles of STZ-NA-induced diabetic rat model. Data are expressed as mean ± S.D. values, calculated using data from three independent experiments per group. Bars labelled with distinct letters show a significant difference (*p* < 0.05). SD: standard drug. PFE: *P. fistulosus* extract.

Transcript quantification revealed that the mRNA expression of GLUT-4 and IRS-1 was markedly downregulated in STZ-NA-treated diabetic control rats compared to the normal control group (*p* < 0.01). Treatment with *P. fistulosus* extract markedly upregulated GLUT-4 and IRS-1 in a dose-dependent manner, with GLUT-4 exhibiting the highest fold change in expression at dose 600 mg/kg (*p* < 0.01), as indicated in Figure 3. These findings were further corroborated by the protein expression of GLUT-4 and IRS-1, revealed via Western blotting. Figure 4 illustrates the effects of *P. fistulosus* extract on protein expression relative to β-actin in skeletal muscles of STZ-NA-induced diabetic rats. While the diabetic control group displayed a significant reduction in protein expression of GLUT-4 and IRS-1, a significant upregulation in the protein expression (*p* < 0.01) of GLUT-4 and IRS-1 was observed among the treatment groups relative to the diabetic control group, with PFE 600 eliciting the maximum response (Figure S1).

### 3.11. Effect of P. fistulosus Extract on the Histopathology of Pancreatic Cells

**Figure 5:**
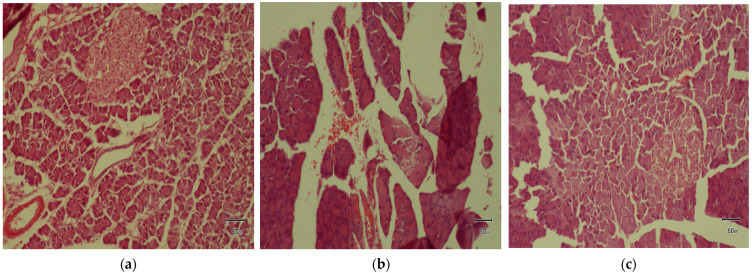

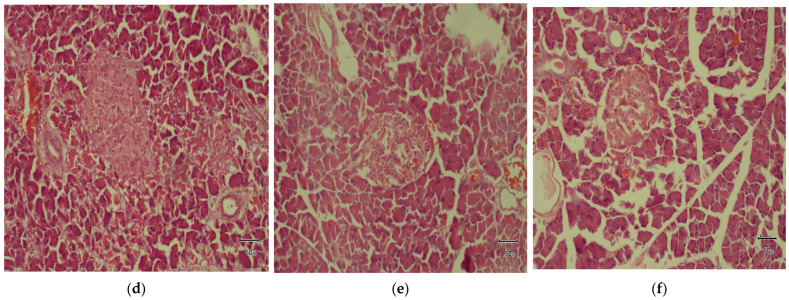
Effect of *P. fistulosus* extract on histopathology of pancreas in STZ-N- induced diabetic rat model. (**a**) Normal control group, (**b**) diabetic control group, (**c**) standard drug group, (**d**) STZ-NA + *P. fistulosus* extract (200 mg), (**e**) STZ-NA + *P. fistulosus* extract (400 mg), and (**f**) STZ-NA + *P. fistulosus* extract (600 mg). Magnification: 10 × 10.

Microscopic examination of the normal control group’s pancreatic tissues revealed typical cellular morphology, exhibiting a highly vascularized and well-preserved lobular arrangement of the pancreatic acini and islets of Langerhans. In contrast, the diabetic control exhibited severe atrophy in pancreatic islets, along with a substantial decline in both the number and size of islets due to β-cell destruction. In addition to marked cellular regression, PFE 200 indicated sparse residual β-cells and extensive amyloid deposition within the islets. Despite mild inflammation, as reflected by congested blood vessels (engorged with RBCs) in the islets, no further considerable pathological alterations were observed in the pancreatic tissues of PFE 400 and PFE 600 treatment groups, as depicted in Figure 5.

## 4. Discussion

Type 2 diabetes mellitus (T2DM) is primarily characterized by persistent hyperglycemia, a relative deficiency in insulin secretion, and resistance, resulting in dysregulated glucose homeostasis [23]. Restoration of normal glucose metabolism can be achieved through mechanisms such as enhancing β-cell regeneration or improving insulin sensitivity in peripheral tissues. Plant-derived bioactive compounds, including flavonoids [24], alkaloids [25], and phenolic acids and terpenoids [26] exhibit antidiabetic properties by modulating key cellular pathways. The STZ-NA model used in this study closely mimics human T2DM by inducing moderate hyperglycemia, β-cell dysfunction, and reduced insulin sensitivity [27]. Additionally, it exhibits responsiveness to sulfonylureas (e.g., glibenclamide) and biguanides (metformin), making it distinct from other experimental models of diabetes [28]. Hence, the current investigation used the STZ-NA model to explore the antidiabetic potential of *Praecitrullus fistulosus* methanolic fruit extract.

In this efficacy trial, body-weight changes among the different rat groups were assessed as a critical measure of the impact of *P. fistulosus* extract on diabetes-induced complications. Diabetic control rats exhibited marked body-weight loss (*p* < 0.05), reflecting glucose metabolism anomalies and muscle atrophy [29,30]. Although the standard drug group has depicted statistically insignificant weight changes, *P. fistulosus* extract stabilized body weights dose-dependently, indicating its protective effect in attenuating diabetes-associated weight loss. These findings are consistent with [31,32], where significant weight reduction in diabetic controls was mitigated by antihyperglycemic plant-based interventions.

In addition to persistent hyperglycemia, the diabetic control group exhibited markedly raised AUC (*p* < 0.05), implying impaired oral glucose tolerance. The glucose tolerance was augmented by the oral administration of *P. fistulosus* extract, as evidenced by the significant reduction in the AUC, proving its promising antihyperglycemic activity, particularly at higher doses. Similar antidiabetic potential of *P. fistulosus* extract has previously been reported against STZ-induced diabetes in a rat model [10]. The underlying mechanism likely involves inhibition of α-glucosidase and α-amylase, delaying intestinal digestion and glucose uptake [33], alongside inhibition of GLUT-4 translocation chiefly due to the strong presence of alkaloids and flavonoids [34]. Comparable glycemic control has been demonstrated by numerous other Cucurbitaceae species via carbohydrase inhibition, improved insulin sensitivity [35], GLP-1 secretion enhancement, and PPAR-γ activation [36].

Serum insulin levels were significantly elevated in *P. fistulosus* extract-treated groups relative to the diabetic control group, largely ascribed to the partial destruction of pancreatic β-cells [37]. It is predominantly driven by the presence of oleanolic acid [33], alkaloids, and phenols, which have an established role in enhancing insulin secretion, insulin sensitivity, and glucose uptake in the peripheral tissues by modulating IRS/PI3K/AKT/FoxO1 and AMPK signaling pathways. Moreover, it activates the MAPK pathway pivotal for β-cells protection and regeneration via oxidative stress reduction [38,39,40]. Comparable results have been documented in turmeric studies using the same diabetes-induction protocol [41]. The homeostasis model HOMA-IR, β-cell function (%B), and insulin sensitivity (%S) are computational frameworks designed to quantify insulin resistance, functioning of pancreatic β-cell, and insulin sensitivity index in pre-clinical and clinical studies [42,43,44]. Although the antidiabetic potential of *P. fistulosus* extract has previously been reported, this is the first study to assess its effect on HOMA-IR, %B, and %S. While earlier studies on *Jatropha gossypifolia* using the same diabetes model observed notable insulin resistance [45], surprisingly, our findings did not reveal such effects in any group. In addition to that, the diabetic control group exhibited significant β-cell dysfunction, which was partially improved by the treatment with *P. fistulosus* extracts. Since STZ-NA co-administration induces a form of T2DM primarily characterized by impaired insulin secretion rather than insulin resistance [46]; hence, these findings were anticipated.

Relative organ weight reflects the physiological and pathological condition of the organ tissues. An increase in relative organ weight is often associated with hyperglycemia-induced hypertrophy due to compensatory growth effects, while a decrease may signify organ degeneration [47]. Our findings indicate no statistically significant differences in the relative kidney and heart weights across all groups, suggesting that these organs remained unaffected. Contrarily, the relative pancreas weight was markedly declined (*p* < 0.05) in the diabetic control group with respect to the normal control group. This has reinforced the findings of previous research that have shown that diabetic rat groups had a decline in pancreas weight primarily due to muscle atrophy [48]. *P. fistulosus* extract, when administered at doses 400 and 600 mg/kg, exhibited a protective effect against oxidative stress and disease progression, as evidenced by the relative pancreas weight that corresponded closely to the standard drug group.

The clinical manifestation of dyslipidemia in diabetes, attributed to insulin insufficiency, insulin resistance, and impaired lipid metabolism, predisposes to cardiovascular diseases [49]. Within this study, the diabetic control group exhibited significant dyslipidemia (*p* < 0.05), characterized by elevated triglycerides, total cholesterol, LDL, and VLDL, alongside reduced HDL. Impaired insulin secretion predominantly accelerates the lipolysis, releasing excess free fatty acids in the blood that transform into triglycerides, LDL, and VLDL [50]. Treatment with metformin and *P. fistulosus* extracts led to improved parameters, with the 600 mg/kg dose showing the most notable effects, significantly reducing the triglycerides and total cholesterol levels, while increasing HDL. This dose-dependent lipid-modulating potential of *P. fistulosus* extract might be linked to the presence of saponins reported to reduce fatty acid synthesis, and it may inhibit the acetyl-CoA cholesterol acyltransferase activity in lipid metabolism and cholesterol absorption [51]. These findings are in agreement with [52], where treatment with curcumin ameliorated the dyslipidemia in comparison to the diabetic group.

Persistent hyperglycemia elevates the reactive oxygen species (ROS) levels, triggering the production of advanced glycation end-products (AGEs) and lipid peroxidation, contributing to oxidative stress, a key factor in T2DM pathogenesis [53]. Malondialdehyde (MDA), a marker of lipid peroxidation, and catalase, an antioxidant enzyme, are primary indicators used to evaluate oxidative damage and antioxidant capacity in diabetic models. According to the current findings, diabetic rats showed significantly higher MDA levels and reduced catalase activity, indicating severe oxidative stress. Similar findings have been reported in analogous models, signifying the redox imbalance leading to diabetes complications [54]. Treatment with *P. fistulosus* extracts and metformin restored these markers in a dose-dependent manner, with dose 600 mg/kg showing the greatest antioxidant effect, nearly normalizing MDA and catalase levels. These findings highlight the strong antioxidative potential of *P. fistulosus* extracts, especially at higher doses. The extract’s potential for enhanced ROS scavenging and stabilization of endogenous antioxidant defenses was previously reinforced by the findings of molecular docking. Notably, α-tocopherol exhibited the highest affinity for α-amylase (−8.2 kcal/mol) through multiple hydrogen bonds, while ursolic acid showed stable binding (−5.6 kcal/mol) [33]. In correspondence with these findings, a marked decline in inflammation mediated by the suppression of inflammatory cytokines was observed in *P. fistulosus* extracts administered groups, which evidenced the strengthened defense against oxidative stress. Previously, the plant’s extract reported antidiabetic and antioxidant efficacy similar to that of gliclazide by reducing lipid peroxide levels and restoring antioxidant activity in pancreatic tissues and plasma, likely ascribed to its secondary metabolites [12,55].

Skeletal muscles, being the primary site for glucose absorption and utilization, exhibit the highest expression of insulin-dependent glucose transporter GLUT-4 and insulin receptor substrate IRS-1, which are known key upstream regulators of the insulin-signaling cascade [56]. Our findings suggest that the mRNA expression of both GLUT-4 and IRS-1 was markedly downregulated in the diabetic control group, with these alterations being further validated at the protein level via Western blotting, particularly due to insulin insufficiency. Insulin deprivation results in reduced IRS-1 and PI3K, and Akt deactivation, subsequently leading to the downregulation of GLUT-4 expression and translocation [57]. However, administration of *P. fistulosus* extract at a dose of 200 mg/kg markedly suppresses this downregulation (*p* < 0.05), with doses of 400 mg/kg and 600 mg/kg resulting in significant upregulation of both mRNA and protein expression. Our study implies that modulation of GLUT-4 and IRS-1 induces a “Domino effect”, attributed to increased insulin secretion, and could be one of the mechanisms of the antidiabetic potential of *P. fistulosus* extract. According to the existing literature, dietary polyphenols have been found to reduce insulin insensitivity by alleviating the insulin-signaling cascade and have a cytoprotective effect on β-cells of the pancreas via the activation of FFAR1 [58].

The histopathological alterations, including pronounced cellular degenerative changes, in the diabetic control group were consolidated by the results of [59]. Although at a dose of 200 mg/kg, histological parameters were marginally improved, significant amyloid deposition, a hallmark feature of advanced T2DM [60] within the islets, was observed. As established in previous findings [61], the administration of *P. fistulosus* extract at higher doses inhibited the irreversible histological variations in pancreatic islets, reinforcing its recuperative effect. This upholds the notion that *P. fistulosus* extract preserves the structural and functional integrity of β-cells. Therefore, the extract’s promising therapeutic potential may stem from a synergistic interplay of its diverse phytochemical constituents. Notwithstanding, these findings provide valuable insights. The pivotal bioactive compounds remained largely uncharacterized, necessitating subsequent investigations in phytochemical profiling and their pharmacological mechanism.

## 5. Conclusions

In a state of prolonged hyperglycemia, oral administration of *P. fistulosus* methanolic extract exhibited strong, dose-dependent antidiabetic potential, as evidenced by improved glycemic control and attenuation of hyperlipidemia in STZ-NA-induced diabetic Wistar rats. Insulin resistance remained unaltered; however, %S and %B improved markedly, reflecting elevated serum insulin levels. Evidence from gene and protein expression analysis further suggests that the extract enhances insulin signaling by upregulating the IRS-1 and GLUT-4. Reduced pancreatic histopathological alterations substantiated a significant reduction of tissue-level oxidative stress indices and cytokine-mediated inflammation. These findings highlight its potential role as an adjunctive therapy for diabetes but necessitate well-designed clinical trials to confirm safety and effectiveness.

## Figures and Tables

**Table 1:** Primer sequence for gene expression profiling.

Genes	(5′-3′) Forward Primer	(5′-3′) Reverse Primer
GLUT-4	“GCTTCTGTTGCCCTTCTGTC”	“TGGACGCTCTCTTTCCAACT”
IRS-1	“GCCAATCTTCATCCAGTTGC”	“CATCGTGAAGAAGGCATAGG”
GAPDH	“TCCCATTCTTCCACCTTTGATGCT”	“ACCCTGTTGCTGTAGCCATATTCAT”

**Table 2:** Effect of *P. fistulosus* extract on body weights during the experimental trial across different rat groups.

Groups	0th Day	30th Day
Normal control	190.3 ± 5.5	235.1 ± 6.2 ^a^
Diabetic control	193.5 ± 6.0	158.2 ± 4.3 ^d^
Standard drug	189.9 ± 5.6	192.7 ± 4.0 ^b^
PFE 200	188.2 ± 5.8	167.3 ± 3.7 ^cd^
PFE 400	185.6 ± 5.9	177.5 ± 4.1 ^bc^
PFE 600	190.7 ± 6.2	189.4 ± 3.6 ^b^

Data are expressed as mean ± S.D. values, calculated using data from six rats per group. Means labelled with distinct letters within columns show a significant difference (*p* < 0.05). PFE: *P. fistulosus* extract.

**Table 3:** Effect of *P. fistulosus* extract on fasting blood glucose levels in STZ-NA-induced diabetic rat model.

Groups	0th Day	30th Day
Normal control	89 ± 2.54 ^e^	91 ± 1.83 ^f^
Diabetic control	289 ± 4.21 ^a^	300 ± 7.37 ^a^
Standard drug	266 ± 5.09 ^cd^	121 ± 2.04 ^e^
PFE 200	271 ± 5.16 ^bc^	209 ± 3.52 ^b^
PFE 400	254 ± 6.85 ^d^	175 ± 2.77 ^c^
PFE 600	280 ± 3.71 ^ab^	138 ± 2.41 ^d^

Data are expressed as mean ± S.D. values, calculated using data from six rats per group. Values are indicated in mg/dL. Means labelled with distinct letters within columns show a significant difference (*p* < 0.05). PFE: *P. fistulosus* extract.

**Table 4:** Effect of *P. fistulosus* extract on insulin resistance, β-cell function, and insulin sensitivity in STZ-NA-induced diabetic rat model.

Groups	Insulin Resistance, HOMA-IR	β-Cell Function (%B)	Insulin Sensitivity (%S)
Normal control	2.94 ± 0.02 ^ab^	166.9 ± 3.57 ^a^	34.2 ± 0.77 ^b^
Diabetic control	3.00 ± 0.17 ^a^	17.3 ± 0.63 ^e^	33.5 ± 0.19 ^b^
Standard drug	2.52 ± 0.02 ^c^	97.6 ± 1.85 ^b^	40.1 ± 1.05 ^ab^
PFE 200	2.60 ± 0.08 ^abc^	34.8 ± 0.71 ^d^	38.5 ± 0.69 ^ab^
PFE 400	2.56 ± 0.01 ^bc^	49.6 ± 0.88 ^d^	40.2 ± 0.83 ^ab^
PFE 600	2.44 ± 0.04 ^c^	75.1 ± 0.42 ^c^	41.6 ± 0.25 ^a^

Data are expressed as mean ± S.D. values, calculated using data from six rats per group. Means labelled with distinct letters within columns show a significant difference (*p* < 0.05). PFE: *P. fistulosus* extract.

**Table 5:** Effect of *P. fistulosus* extract on relative organ weights during the experimental trial across different rat groups.

Groups	Relative Kidney Weight (%)	Relative Heart Weight (%)	Relative Pancreas Weight (%)
Normal control	0.74 ± 0.02	0.34 ± 0.02	0.64 ± 0.05 ^a^
Diabetic control	0.76 ± 0.06	0.37 ± 0.04	0.44 ± 0.01 ^c^
Standard drug	0.77 ± 0.03	0.35 ± 0.03	0.61 ± 0.03 ^a^
PFE 200	0.72 ± 0.05	0.36 ± 0.09	0.52 ± 0.06 ^bc^
PFE 400	0.75 ± 0.04	0.33 ± 0.03	0.58 ± 0.02 ^ab^
PFE 600	0.73 ± 0.07	0.34 ± 0.05	0.60 ± 0.03 ^ab^

Data are expressed as mean ± S.D. values, calculated using data from six rats per group. Means labelled with distinct letters within columns show a significant difference (*p* < 0.05). PFE: *P. fistulosus* extract.

**Table 6:** Effect of *P. fistulosus* extract on lipid profile in STZ-NA-induced diabetic rat model.

Groups	TG	TC	HDL	LDL	VLDL
Normal control	108 ± 2.2 ^e^	120 ± 1.7 ^d^	42 ± 1.1 ^a^	56 ± 2.5 ^e^	22 ± 1.7 ^e^
Diabetic control	159 ± 3.4 ^a^	173 ± 3.3 ^a^	16 ± 1.5 ^c^	125 ± 4.4 ^a^	32 ± 2.4 ^a^
Standard drug	112 ± 1.1 ^de^	154 ± 2.1 ^cd^	30 ± 1.6 ^b^	74.6 ± 2.3 ^d^	22 ± 2.3 ^de^
PFE 200	146 ± 2.9 ^b^	139 ± 2.4 ^b^	24 ± 2.3 ^b^	101 ± 3.6 ^b^	29 ± 1.6 ^b^
PFE 400	125 ± 2.5 ^c^	125 ± 3.2 ^c^	26 ± 3.7 ^b^	88 ± 1.1 ^c^	25 ± 1.8 ^c^
PFE 600	121 ± 2.1 ^cd^	127 ± 1.8 ^d^	29 ± 1.2 ^b^	72 ± 1.8 ^d^	24 ± 2.1 ^cd^

The data of the lipid profile are presented in mg/dL. Mean ± S.D. values were calculated using data from six rats per group. Means labelled with distinct letters within columns show a significant difference (*p* < 0.05). PFE: *P. fistulosus* extract.

**Table 7:** Effect of *P. fistulosus* extract on MDA and catalase.

Groups	MDA (μmol/L)	Catalase (KU/L)
Normal control	1.11 ± 0.08 ^f^	1.71 ± 0.07 ^a^
Diabetic control	2.90 ± 0.15 ^a^	0.40 ± 0.08 ^f^
Standard drug	1.61 ± 0.08 ^d^	1.41 ± 0.10 ^c^
PFE 200	2.31 ± 0.09 ^b^	0.90 ± 0.07 ^e^
PFE 400	1.90 ± 0.06 ^c^	1.10 ± 0.06 ^d^
PFE 600	1.40 ± 0.06 ^e^	1.51 ± 0.11 ^b^

Data are expressed as mean ± S.D. values, calculated using data from six rats per group. Means labelled with distinct letters within columns show a significant difference (*p* < 0.05). PFE: *P. fistulosus* extract.

**Table 8:** Effect of *P. fistulosus* extract on TNF-α and IL-6.

Groups	TNF-α (pg/mL)	IL-6 (pg/mL)
Normal control	45.02 ± 3.85 ^e^	28.50 ± 1.38 ^e^
Diabetic control	161.1 ± 6.20 ^a^	93.33 ± 4.63 ^a^
Standard drug	75.11 ± 3.06 ^d^	47.00 ± 3.41 ^d^
PFE 200	118.66 ± 3.27 ^b^	80.83 ± 2.93 ^b^
PFE 400	93.00 ± 2.37 ^c^	65.04 ± 2.10 ^c^
PFE 600	72.16 ± 2.64 ^d^	51.5 ± 1.87 ^d^

Data are expressed as mean ± S.D. values, calculated using data from six rats per group. Means labelled with distinct letters within columns show a significant difference (*p* < 0.05). PFE: *P. fistulosus* extract.

## Data Availability

The original contributions presented in the study are included in the article/Supplementary Materials, further inquiries can be directed to the corresponding author.

## Supplementary Materials

The following supporting information can be downloaded at https://www.mdpi.com/article/10.3390/foods14213764/s1, Figure S1: Unprocessed Western Blots for Figure 4.

## Abbreviations

The following abbreviations are used in this manuscript:

T2DM	Type 2 diabetes mellitus
STZ-NA	Streptozotocin–nicotinamide
ELISA	Enzyme-linked immunosorbent assay
OGTT	Oral glucose-tolerance test
AUC	Area under the curve
HOMA-IR	Homeostatic Model Assessment of Insulin Resistance
TC	Total cholesterol
TG	Triglycerides
HDL	High-density lipoprotein
LDL	Low-density lipoprotein
VLDL	Very low density lipoprotein
MDA	Malondialdehyde
CAT	Catalase
TNF-α	Tumor necrosis factor alpha
IL-6	Interleukin 6
ECL	Enhanced chemiluminescence
ANOVA	Analysis of variance
BGL	Blood glucose level
%B	β-cell function
%S	Insulin sensitivity
TIDM	Type 1 diabetes mellitus
ROS	Reactive oxygen species
AGEs	Advanced glycation end-products
