# Supplementary figures and images for: Praecitrullus fistulosus Extract Exhibits Antidiabetic Potential by Augmenting Insulin-Signaling Cascade, GLUT-4 and IRS-1, in Streptozotocin–Nicotinamide-Induced Diabetic Rats
Source: Foods. 2025 Nov 3;14(21):3764. doi: 10.3390/foods14213764 (PMC12609042; doi:10.3390/foods14213764)

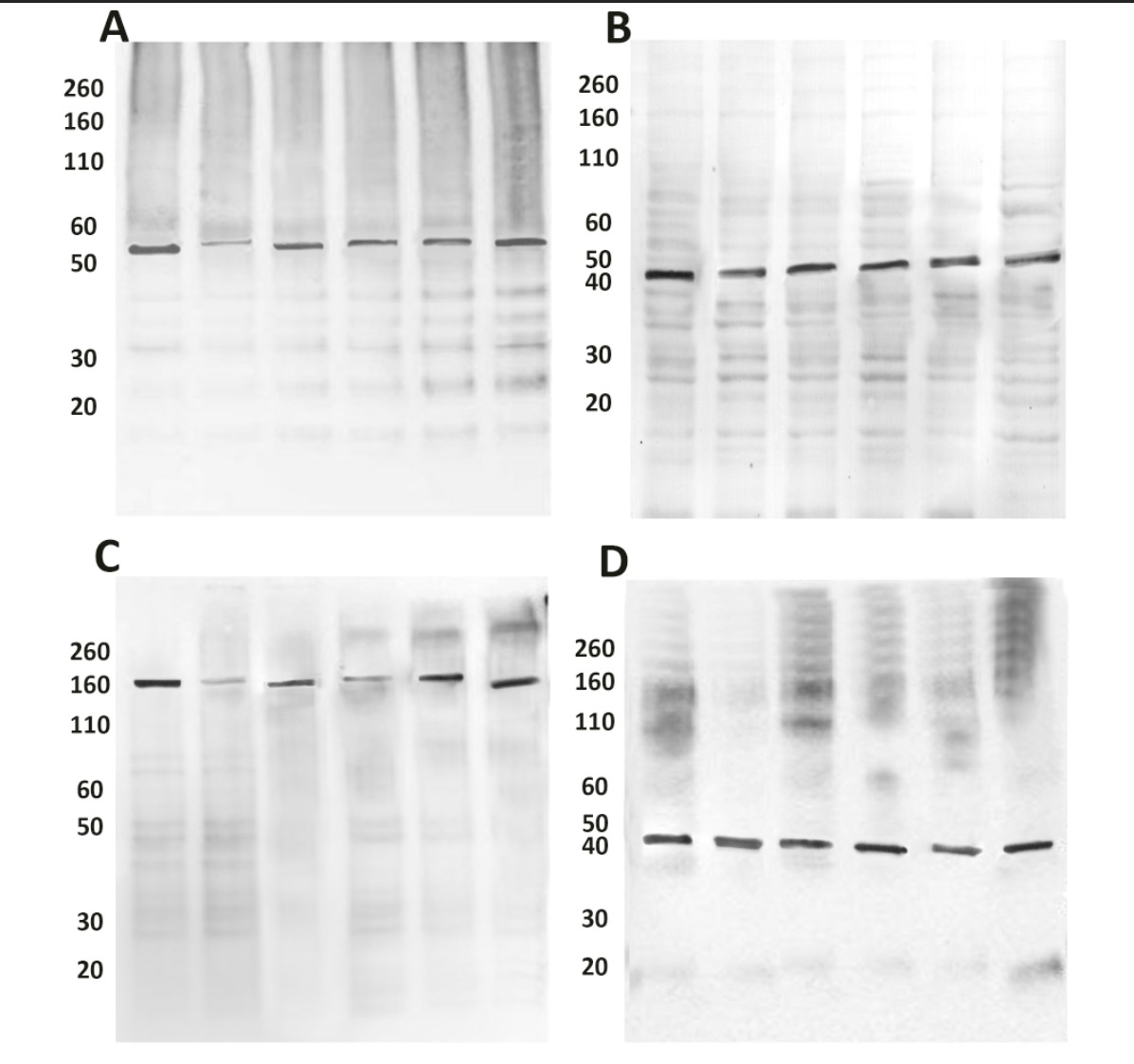

Supplement: Supplementary file 1 [file foods-14-03764-s001.zip › foods-3869593-supplementary.jpeg]
